# Studies on Age-Related Changes in Equine Cheek Teeth Angulation and Dental Drift

**DOI:** 10.3389/fvets.2021.804061

**Published:** 2022-02-15

**Authors:** Tiziana Liuti, Carola R. Daniel, Padraic Martin Dixon, Richard J. M. Reardon

**Affiliations:** ^1^Royal (Dick) School of Veterinary Studies, The University of Edinburgh, Edinburgh, United Kingdom; ^2^Independent Researcher, Edinburgh, United Kingdom

**Keywords:** horse, anatomy, diastema, drift, angulation

## Abstract

**Background:**

Cheek teeth (second through fourth premolars and first through third molars) diastema is a common and painful equine disorder caused by the absence of effective tight interdental contact between these teeth. Limited objective information is available on the angulation of equine cheek teeth that control dental drift or on mesial or distal equine cheek teeth drift that should normally prevent this disorder.

**Objectives:**

To measure the angulation of the mesial and distal cheek teeth in horses of different ages, quantify age-related cheek teeth mesial and distal dental drift, and measure the cheek teeth row length in horses of different ages.

**Study Design:**

Retrospective review of computed tomographic images of equine heads.

**Methods:**

Case details and CT images from clinical equine cases that had undergone standing CT head examination were collated.

Three sets of measurements were acquired from each head. “Head size” calculated as the distance between the caudal aspect of the orbit and the caudal aspect of the naso-incisive notch was used to standardize measurements in different sized heads. The length of the cheek teeth rows measured from the mesial aspect of the Triadan 06 occlusal surface to the distal aspect of the Triadan 11 occlusal surface. The rostro-caudal (antero-posterior) position and angulation of the mandibular and maxillary Triadan 06 and 11 teeth were measured in relation to reference lines drawn on CT images.

**Results:**

Significant mesial drift occurred in the maxillary and mandibular Triadan 11s. Despite their distal angulation, the upper and lower Triadan 06s also drifted mesially. The mean angulation of Triadan 06 and 11 mandibular teeth (17.8 and 26.2°, respectively) was almost double that of maxillary teeth (9.2 and 13.3°, respectively) with both Triadan 11s having greater angulation than the 06s. Cheek teeth angulation only significantly decreased in the mandibular 06s. Cheek teeth arcade lengths decreased with age, but these decreases were not significant.

**Main Limitations:**

Limitations include the relatively small sample size.

**Conclusions:**

In the population of horses used for this study, age related mesial drift occurred in both Triadan 06 and 11s, and the angulation of these teeth did not decrease with age in most arcades.

## Introduction

Mammalian teeth within a complete dental arch normally drift in a mesial direction (toward the midline, i.e., between the first incisor teeth) with age. This normal, posteruptive dental movement is to compensate for normal interproximal dental wear at contact areas of adjacent teeth and so prevent abnormally wide interproximal spaces (diastemata) from developing. However, equine teeth do not form a continuous arch. Drift in the anteroposterior plane only, occurs in their straight row of six cheek teeth (i.e., second through fourth premolars and the three molar teeth) and equine mesial cheek teeth drift has been described as rostral drift (1–3).

Mesial drift is additionally needed by equine teeth because their long (clinical and reserve) crowns taper in toward their apices, thus making the clinical crowns in older horses progressively smaller in a mesio-distal direction following normal eruption and occlusal wear. This feature makes equine teeth more prone to developing diastemata with age (termed *senile diastemata*) (2).

The mechanisms of posteruption dental movement are complex and incompletely understood in all mammals. They include direct (approximal) pressure from adjacent teeth, vertical masticatory forces on teeth that are inclined (angulated, tipped) that causes anterior (mesial, rostral) or posterior (distal, caudal) teeth movement of the angulated and adjacent teeth, depending on their inclination. Pressure from the cheeks and tongue and contraction of transeptal fibers also play a part in dental movement in brachydont species (4–8).

In horses, the prolonged eruption of the mesially (rostrally) inclined clinical crowns [with distally (caudally) angulated reserve crowns] of the caudal cheek teeth, especially the Triadan 11 (third molar), directly forces the cheek teeth row in an anterior direction causing a mesial drift (2). In addition, mesial directed forces caused by occlusal pressures on these inclined teeth, also contributes to the mesial drift. A computed tomographic (CT) study showed equine third maxillary molars (Triadan 11) clinical crowns to have a mean mesial drift of 24.8 mm between horses under 6 and over 16 years of age (3).

As noted, equine teeth do not form a complete arch due to the presence of an (edentulous) interdental space (previously termed a *physiological diastema*) between their incisor and premolar teeth, and a necessary adaption is that the clinical crowns of the second premolar teeth (Triadan 06s) are inclined distally (caudally). These inclined 06s directly exert a caudally (distally) directed (approximal) pressure on the adjacent Triadan 07s and, thus, indirectly on the other cheek teeth. Additionally, as described earlier, masticatory pressures also exert a caudally directed force on these distally inclined teeth. A combination of the distally directed forces of the rostral cheek teeth in combination with mesial drift of the caudal cheek teeth normally keeps the six equine cheek teeth tightly compressed together occlusally.

Both mesial and distal equine cheek teeth drift have been demonstrated experimentally following extraction of maxillary 08s that showed marked narrowing of the extraction space (at a rate of 39–41% of the extraction site mesio-distal length per year) along with inclination of the clinical crowns of the remaining teeth into the extraction space from *both* directions (9, 10). Similarly, Townsend et al. show mesial and distal dental drift into the extraction sites of 50 clinical equine cases following cheek teeth exodontia with a mean closure of extraction sites of 16% (range 4–50%) per year along with inclination of all remaining teeth toward the extraction sites (11).

Studies in rats, which also have an interdental space between their incisor and molar teeth (rats have no premolars) show that dental drift into the empty alveolus following extraction of a molar is largely in a mesial direction (5, 6).

Cheek teeth diastemata with resultant periodontal disease is a very common equine dental disorder. A UK general practice study showing a prevalence of 50% in the general equine population (12). This disorder was also regarded as the most common, painful equine dental disorder in equine referral clinic studies (13, 14). The etiopathogenesis of this disorder is incompletely understood, but the presence of effective dental drift (in both directions) should bring and dynamically maintain the occlusal aspects of cheek teeth in tight occlusal contact and, thus, prevent or treat this disorder.

Interproximal dental wear due to individual movement of adjacent teeth and tapering of the cheek teeth crowns should cause equine cheek teeth rows to become shorter with age, but this feature does not appear to have been documented in horses.

No study has attempted to quantified distal (caudal) drift of equine maxillary or mandibular cheek teeth or mesial drift of equine mandibular cheek teeth using CT. This drift is largely governed by the angulation of teeth at the peripheries of the cheek teeth row. The curvature and position of the equine cheek teeth in horses of different ages was radiographically evaluated by Huthmann et al. (15). The maxillary cheek teeth curvature changed minimally with increasing age, but the mesio-occlusal angle of the maxillary cheek teeth did increase with age. There were less significant changes in the mandibular cheek teeth arcades (15).

A better understanding of equine cheek teeth drift may help explain why cheek teeth diastemata develops in some horses and may also indicate how this disorder may be prevented or treated.

The aim of this study was to quantify cheek teeth angulation in the mesio-distal plane, quantify mesial and distal mandibular and maxillary cheek teeth drift, and compare cheek teeth row length in horses of different ages using CT.

## Materials and Methods

This study was approved by the Ethical Committee of the University of Edinburgh Veterinary School (52.21). Case details and CT images from clinical equine cases that had undergone standing CT head examination between January 1, 2011, and December 31, 2020, at R(D)SVS were collated.

### CT Examination

All CT studies were undertaken using standardized protocols optimized for the equine head. During the study period, two different CT scanners were used: from 2011 to 2016 a four-row MDCT unit (Somatom® Volume Zoom, Siemens, Germany) with 3 mm slice thickness, 120 kVp, 100 mA, matrix 512 × 512, pitch 1 was used. The later cases were scanned with a 64-row MDCT scanner (Somatom® Definition AS Siemens, Erlangen, Germany) with slice thickness 0.6 mm, 120 kVp, 150 mA, matrix 512 × 512, pitch of 1.0.

### Case Selection

CT Images [bone window (H70)] were reviewed by a diagnostic imaging resident and diplomate using a computer workstation (Apple Mac Pro, Apple, USA) with a calibrated LCD flat screen monitor, using a dedicated DICOM viewer software (Horos, Purview, Annapolis MD, USA, version 3.3.6).

Cases with CT evidence of any of the following: motion artifact during image acquisition, missing or supernumerary teeth, cheek teeth diastemata, displaced or fractured cheek teeth, or craniofacial abnormalities (e.g., brachygnathism or prognathism) were excluded from the study.

### CT Measurements

Three sets of measurements were acquired for this study. Sagittal plane CT reconstructions were used to make measurements using the calibrated measuring tools in DICOM viewer software.

**Figure 1:**
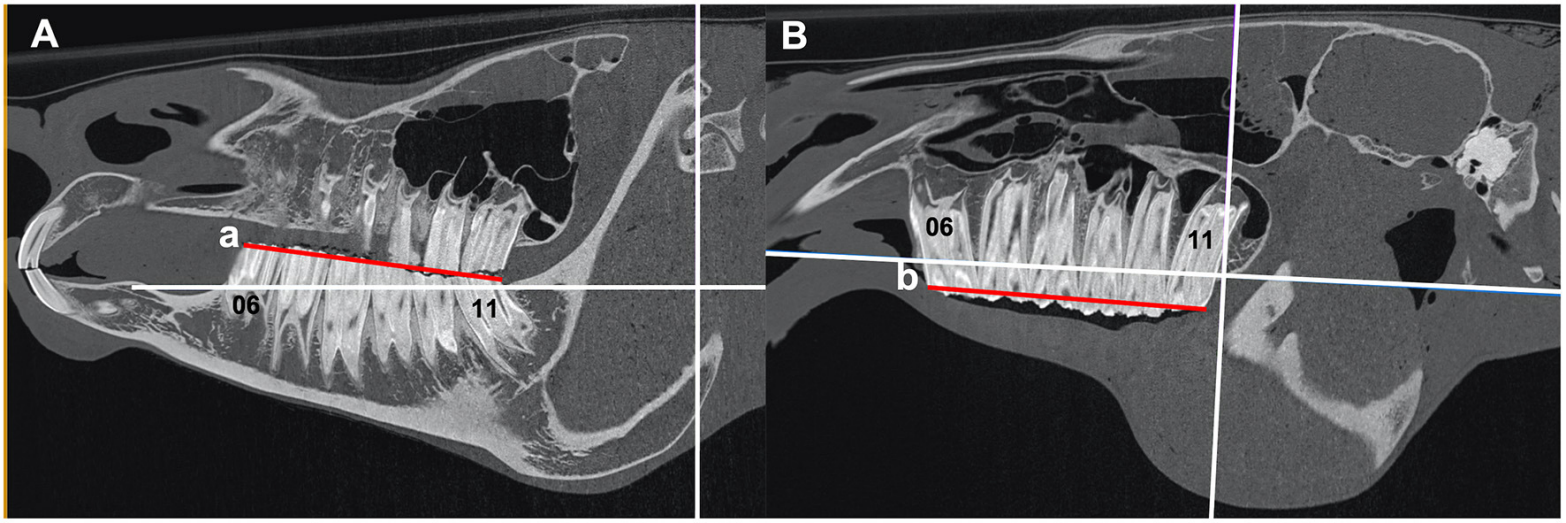
**(A,B)** Sagittal CT images of an equine head showing the length (red lines) of the mandibular (a) and maxillary (b) arcades measured at the occlusal surface from the mesial aspect of 06 to the distal aspect of 11. The red line “a” and “b” shows the length of the mandibular and maxillary arcade, respectively.

1) *Head size*
  The distance between the caudal aspect of the orbit and the caudal aspect of the naso-incisive notch was measured in all cases and was used as an estimate of head size.
2) *The length of the cheek teeth rows*
  Measured from the mesial aspect of the Triadan 06 occlusal surface to the distal aspect of the Triadan 11 occlusal surface (Figures 1A,B).
3) *The rostro-caudal (antero-posterior) position and angulation of the mandibular and maxillary Triadan 06 and 11 teeth*
  Measured in relation to reference lines drawn on CT images.

### 3a) Mandibular Teeth

**Figure 2:**
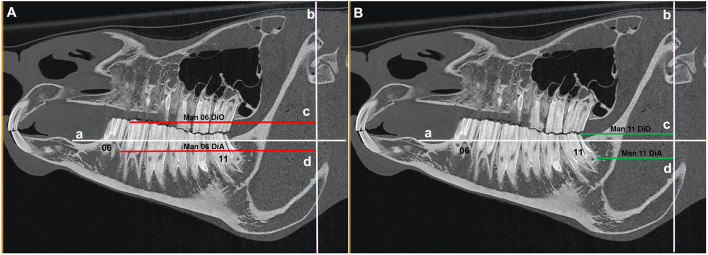
**(A)** Sagittal CT image of an equine head showing line “a” that intersects the dorsal aspect of the mandibular bone immediately mesial to the mandibular second premolar (Triadan 06) tooth and the dorsal aspect of the mandibular bone immediately distal to the mandibular third molar (Triadan 11) tooth and line “b” at the level of the rostral aspect of the mandibular condyle that lies perpendicular to line “a.” The red lines “c” (Man 06 DiO) and “d” (Man 06 DiA) run from the line “b” to the distal aspect of the occlusal surface of Triadan 06 and to the distal aspect of the apex of Triadan 06, respectively. **(B)** Sagittal CT image of an equine head showing line “a” that intersects the dorsal aspect of the mandibular bone immediately mesial to the mandibular second premolar (Triadan 06) tooth and the dorsal aspect of the mandibular bone immediately distal to the mandibular third molar (Triadan 11) tooth and line “b” at the level of the rostral aspect of the mandibular condyle that lies perpendicular to line “a.” The green lines “c” (Man 11 DiO) and “d” (Man 11 DiA) run from the line “b” to the distal aspect of the occlusal surface of Triadan 11 and to the distal aspect of the apex of Triadan 11, respectively.

**Figure 3:**
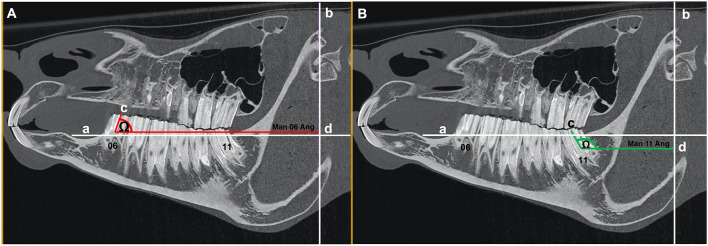
**(A)** Sagittal CT image of an equine head showing line “a” that intersects the dorsal aspect of the mandibular bone immediately mesial to the mandibular second premolar (Triadan 06) tooth and the dorsal aspect of the mandibular bone immediately distal to the mandibular third molar (Triadan 11) tooth and line “b” at the level of the rostral aspect of the mandibular condyle that lies perpendicular to line “a.” The red line “c” drawn through the central long axis of mandibular Triadan 06 intersects line “d” that lies parallel to line “a” and perpendicular to line “b.” The angle (Ω) created with the intersection of line “c” and line “d” was measured (Man 06 Ang). **(B)** Sagittal CT image of an equine head showing line “a” that intersects the dorsal aspect of the mandibular bone immediately mesial to the mandibular second premolar (Triadan 06) tooth and the dorsal aspect of the mandibular bone immediately distal to the mandibular third molar (Triadan 11) tooth and line “b” at the level of the rostral aspect of the mandibular condyle that lies perpendicular to line “a.” The green line “c” drawn through the central long axis of mandibular Triadan 11 intersects line “d” that lies parallel to line “a” and perpendicular to line “b.” The angle (Ω) created with the intersection of line “c” and line “d” was measured (Man 11 Ang).

For each mandibular cheek teeth row, two reference lines were drawn:

a. A line intersecting the dorsal aspect of the mandibular bone immediately mesial to the mandibular second premolar (Triadan 06) tooth and the dorsal aspect of the mandibular bone immediately distal to the mandibular third molar (Triadan 11) tooth (Line “a”) (Figures 2A,B, 3A,B).
b. A line at the level of the rostral aspect of the mandibular condyle that lay perpendicular to line “a” (Line “b”) (Figures 2A,B, 3A,B).

#### Antero-Posterior (Rostro-Caudal) Position of Teeth

Distances from reference line “b” to the distal aspects of the mandibular Triadan 06 and 11 teeth were measured at the most occlusal and apical aspects of the Triadan 06 and 11 teeth (red and green lines “c” and “d”) (Man 06 DiO, Man 06 DiA, Man 11 DiO, Man 11 DiA) (Figures 2A,B).

#### Angles of Triadan 06 and 11 Teeth

Lines of best fit were drawn along the central long axis extending from the occlusal surface to the reserve crowns of the mandibular Triadan 06 and 11 teeth, and the angle of these lines in relation to line “b” were calculated (Man 06 Ang and Man 11 Ang) (Figures 3A,B).

### 3b) Maxillary Teeth

**Figure 4:**
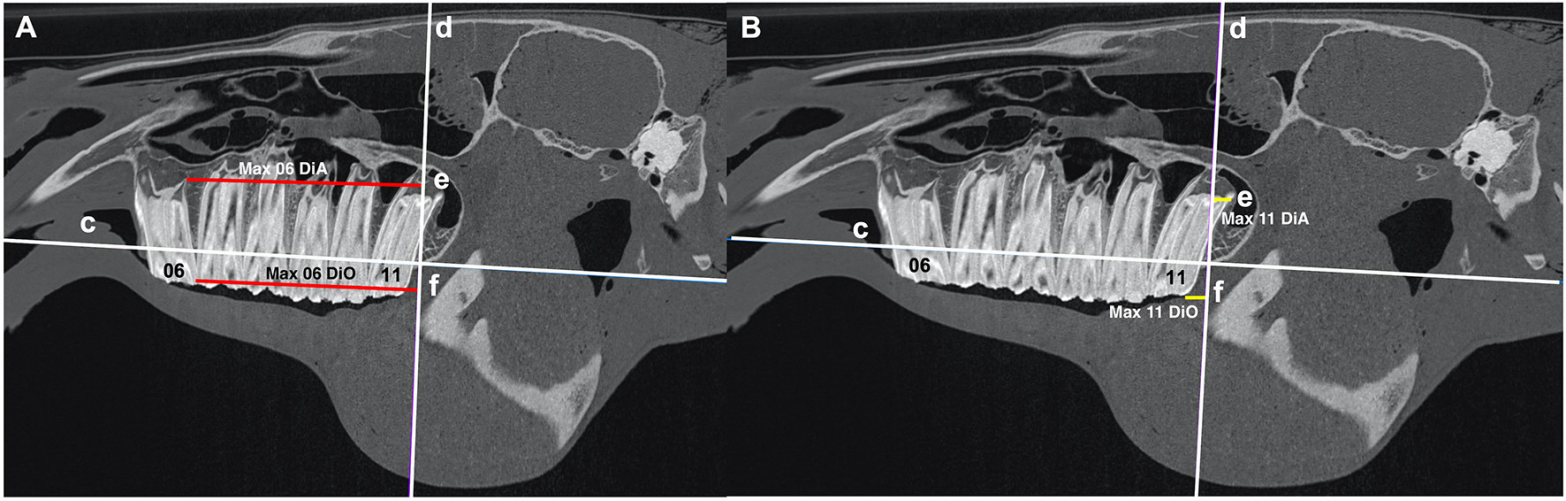
**(A)** Sagittal CT image of an equine head showing line “c” that intersects the dorsal aspect of the maxillary bone immediately mesial to the maxillary second premolar (Triadan 06) tooth and the dorsal aspect of the maxillary bone immediately distal to the maxillary third molar (Triadan 11) tooth and line “d” at the level of the rostral orbit that lies perpendicular to line “c.” The red lines “e” (Max 06 DiA) and “f” (Max 06 DiO) run from line “d” to the distal aspect of the occlusal surface of Triadan 06 and to the distal aspect of the apex of Triadan 06, respectively. **(B)** Sagittal CT image of an equine head showing line “c” that intersects the dorsal aspect of the maxillary bone immediately mesial to the maxillary second premolar (Tridan 06) tooth and the dorsal aspect of the maxillary bone immediately distal to the maxillary third molar (Triadan 11) tooth and line “d” at the level of the rostral orbital rim that lies perpendicular to line “c.” The yellow lines “e” (Max 11 DiA) and “f” (Max 11 DiO) run from the line “d” to the distal aspect of the occlusal surface of Triadan 11 and to the distal aspect of the apex of Triadan 11, respectively.

**Figure 5:**
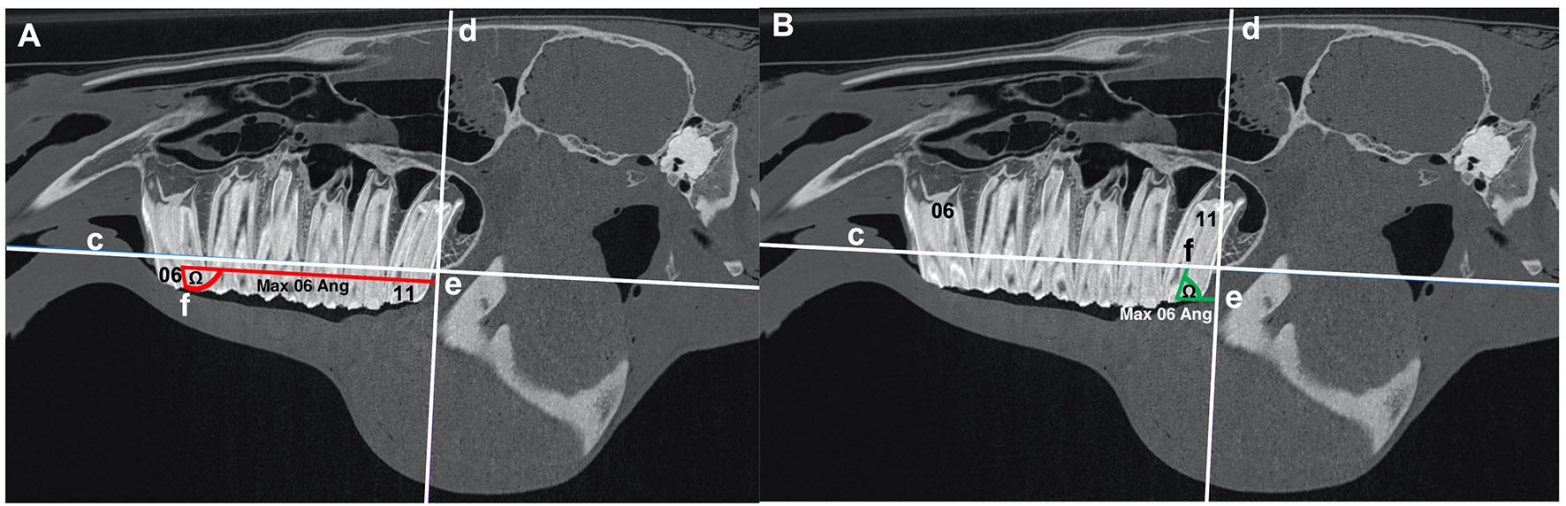
**(A)** Sagittal CT image of an equine head showing line “c” that intersects the dorsal aspect of the maxillary bone immediately mesial to the maxillary second premolar (Triadan 06) tooth and the dorsal aspect of the maxillary bone immediately distal to the maxillary third molar (Triadan 11) tooth. Line “d” is at the level of the rostral orbit and lies perpendicular to line “c.” The red line “f” drawn through the central long axis of maxillary Triadan 06 intersects line “e” that lies parallel to line “c” and perpendicular to line “d.” The angle (Ω) created by the intersection of line “f” and line “e” was measured (Max 06 Ang). **(B)** Sagittal CT image of an equine head showing line “c” that intersects the dorsal aspect of the maxillary bone immediately mesial to the maxillary second premolar (Tridan 06) tooth and the dorsal aspect of the maxillary bone immediately distal to the maxillary third molar (Triadan 11) tooth. Line “d” at the level of the rostral orbit lies perpendicular to line “c.” The green line “f” drawn through the central long axis of the maxillary Triadan 11 intersects line “e” that lies parallel to line “c” and perpendicular to line “d.” The angle (Ω) created by the intersection of line “f” and line “e” was measured (Max 11 Ang).

For each maxillary cheek teeth row, two reference lines were drawn as previously described (3).

a. A line intersecting the ventral aspect of the maxillary bone immediately mesial to the maxillary Triadan 06 tooth to the dorsal aspect of the mandibular bone immediately distal to the mandibular Triadan 11 tooth (Figures 4A,B, 5A,B).
b. A line perpendicular to line “c” at the level of the rostral aspect of the orbit (Figures 4A,B, 5A,B).

#### Antero-Posterior (Rostro-Caudal) Position of Teeth

Distances from reference line “d” to the distal aspects of the maxillary Triadan 06 and 11 teeth were measured at the most occlusal and apical aspects of these teeth (Max 06 DiO, Max 06 DiA, Max 11 DiO, Max 11 DiA). Measurements rostral to line “d” were positive and those caudal to it were negative (Figures 4A,B).

#### Angles of Teeth

Lines of best fit were drawn along the central long axis extending from the occlusal surface to the reserve crowns of the maxillary Triadan 06 and 11 teeth and the angle of these lines in relation to line “d” were calculated (Max 06 Ang and Max 11 Ang) (Figures 5A,B).

### Statistical Analyses

The mean of left- and right-sided measures were used for analyses. Linear and dental angle measures and horse ages were assessed for normality by graphical assessment and using Shapiro–Wilks tests, the results of which were used to guide appropriate statistical test choice (i.e., parametric or non-parametric).

To allow comparison of tooth positions and cheek teeth row lengths between ages while accounting for differences in head sizes, adjusted rostro-caudal position measures, and adjusted row lengths were calculated (“adjusted measures”) by scaling to the mean measured head size of the samples.

For example, in a case with head size 10% smaller than the mean size, tooth position measures were increased by 10%.

Dental angle measures were not adjusted for head size.

The associations between horse age and “adjusted measures” as well as dental angle were compared using Pearson correlation and simple linear regression. Statistical analyses and production of graphs were performed in RStudio™. Significance was set at *P* < 0.05.

To allow comparisons with a previous study Liuti et al. (3) mean values were calculated for measures between age groups: <6, 6–15, and >15 years.

## Results

A total of 67 cases were included in this study based on the above inclusion criteria with 291 cases excluded having not met the inclusion criteria. There were 23 cases from 2011 to 2016 and 44 cases from 2017 to 2020 of mean age 10.8 years (SD: 4.1; range: 3–21 years) and included 24 females and 43 males. Breed distribution is shown in Table 1. The main reasons for these CT head examinations included suspected cheek teeth apical infection, head shaking, ataxia, and head trauma.

**Table 1:** Breed distribution in the study population.

**Breed**	**Number**
Thoroughbred and crosses	26
Sports horses	14
Warmblood	8
Irish Draft and crosses	3
Crossbreed	3
Welsh Cob	3
Welsh Pony	3
Connemara	2
Arabian	1
Clydesdale cross	1
Highland pony	1
Pony	1
Shetland Pony	1

Mean measures (absolute and adjusted) of distance and mean angles for each site as well as cheek teeth row lengths are shown in Table 2. Mean values for measures subdivided by different age groups are shown in Table 3.

**Table 2:** Mean measures (cm) and angles (degrees) and adjusted measures for each site from 67 horses.

**Site**	**Mean measure cm (SD)**	**Mean adjusted measure cm (SD)**	**Association with age**	
			**Pearson correlation**	**Lin reg *p*-value**
**Maxillary**				
Max 06 DiA	15.6 (1.6)	15.4 (1.0)	0.22	0.080
Max 06 DiO	14.5 (1.5)	14.4 (1.1)	0.32	**0.009**
Max 06 Ang	80.8 (5.1)		−0.08	0.522
Max 11 DiA	−0.3 (1.1)	−0.4 (1.1)	0.50	**<0.001**
Max 11 DiO	0.5 (0.9)	0.5 (0.9)	0.41	**<0.001**
Max 11 Ang	103.3 (4.7)		0.18	0.138
Arcade Length	19.0 (1.6)	18.8 (1.4)	−0.14	0.345
**Mandible**				
Man 06 DiO	27.9 (2.1)	27.7 (2.0)	0.26	**0.034**
Man 06 DiA	29.4 (2.1)	29.3 (2.0)	0.14	0.258
Man 06 Ang	72.2 (4.4)		0.37	**0.002**
Man 11 DiO	13.4 (1.4)	13.3 (1.4)	0.37	**0.001**
Man 11 DiA	11.5 (1.5)	11.4 (1.5)	0.46	**<0.001**
Man 11 Ang	116.2 (5.1)		−0.18	0.136
Arcade length	19.2 (1.7)	19.0 (1.6)	−0.21	0.095

*Results of comparisons of angles and adjusted measures with age shown as Pearson correlation coefficients, and P-values generated from simple linear regression (Lin reg). Significant associations shown in bold. Sites: Max, maxillary; Man, mandibular; DiA, Disto-Apical; DiO, Disto-Occlusal; Ang, Angle; numbers, Triadan tooth position*.

**Table 3:** Mean adjusted measures (cm) and angles (degrees) for each site from 67 horses subdivided by age groups.

**Site**	Mean adjusted^*^	Mean adjusted^*^measure (SD)		
	**measure cm (SD)**			
		**Age group 1**	**Age group 2**	**Age group 3**
		**(*n* = 5)**	**(*n* = 51)**	**(*n* = 11)**
		** <6 years**	**6–15 years**	**>16 years**
**Maxillary**				
Max 06 DiA	15.4 (1.0)	14.6 (0.4)	15.4 (1.1)	15.8 (0.8)
Max 06 DiO	14.4 (1.1)	13.5 (0.6)	14.4 (1.1)	15.0 (0.8)
Max 06 Ang	80.8 (5.1)	81.1 (5.3)	81.0 (5.1)	79.8 (5.3)
Max 11 DiA	−0.4 (1.1)	−1.5 (1.1)	−0.6 (0.8)	1.1 (0.9)
Max 11 DiO	0.5 (0.9)	−0.3 (0.8)	0.4 (0.8)	1.5 (0.8)
Max 11 Ang	103.3 (4.7)	100.7 (4.7)	103.2 (4.3)	105.4 (6.1)
Arcade Length	18.8 (1.4)	18.6 (1.3)	19.0 (1.5)	17.9 (1.1)
**Mandible**				
Man 06 DiO	27.7 (2.0)	27.0 (0.8)	27.6 (2.0)	28.3 (2.0)
Man 06 DiA	29.3 (2.0)	29.1 (0.8)	29.3 (2.1)	29.5 (1.9)
Man 06 Ang	72.2 (4.4)	69.1 (2.8)	71.9 (4.2)	75.3 (4.6)
Man 11 DiO	13.3 (1.4)	12.7 (1.2)	13.2 (1.4)	14.2 (1.3)
Man 11 DiA	11.4 (1.5)	10.0 (1.0)	11.3 (1.3)	12.6 (1.9)
Man 11 Ang	116.2 (5.1)	115.7 (4.9)	116.8 (5.2)	113.9 (4.9)
Arcade length	19.0 (1.6)	19.6 (1.2)	19.1 (1.6)	18.2 (1.2)

*Sites: Max, maxillary; Man, mandibular; DiA, Disto -Apical; DiO, Disto -Occlusal; Ang, Angle; numbers, Triadan tooth position*.

* *Angles not adjusted – shaded gray*.

The Triadan 06 teeth clinical crowns were angled (inclined) distally, and the Triadan 11 teeth clinical crowns were inclined mesially with, on average, slightly more angulation in the mandibular teeth, which could be observed in the difference between the occlusal and apical rostro-caudal measures and by the angle measurements (Table 2).

### Associations Between Measures and Age

**Figure 6:**
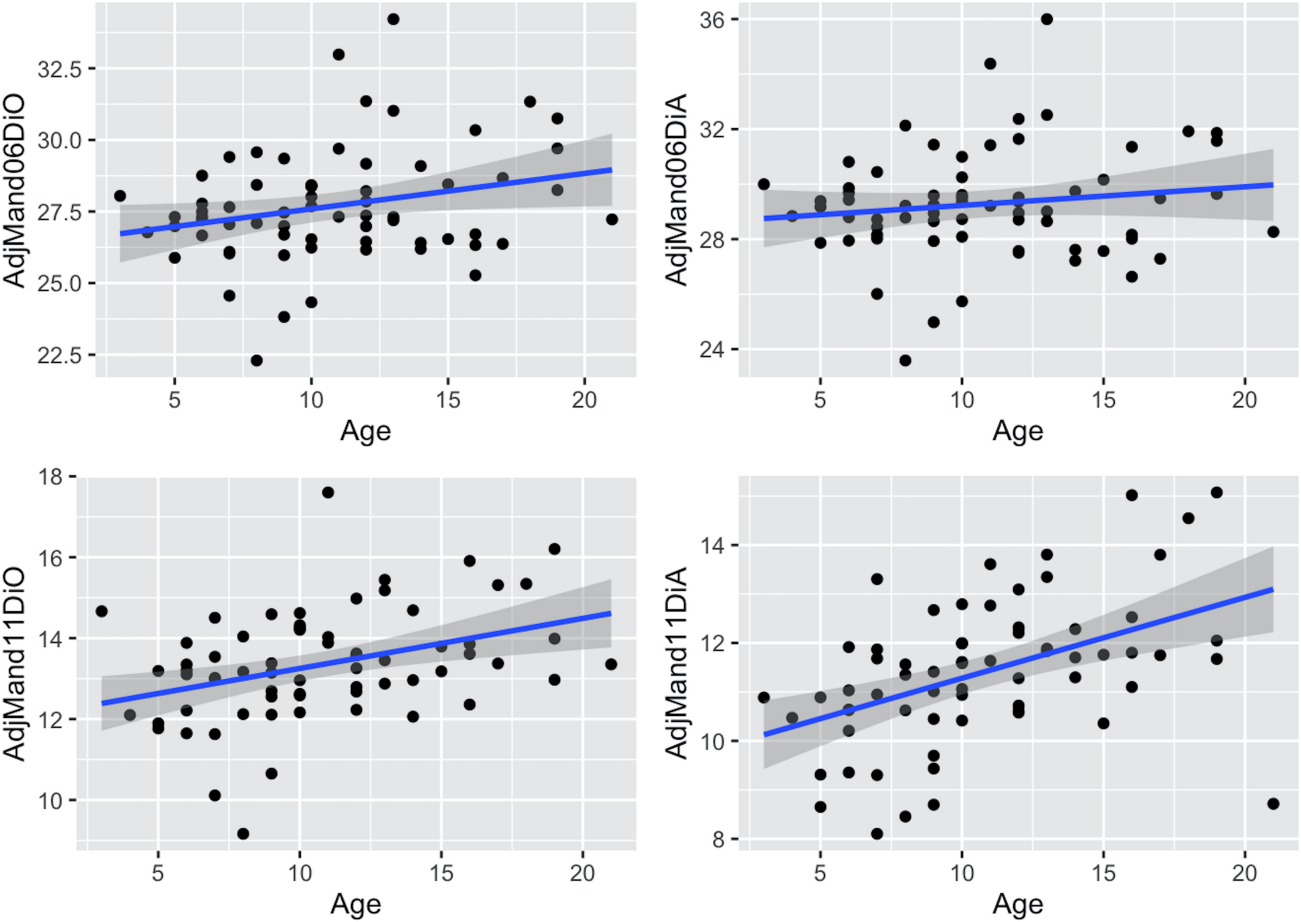
Scatterplots with linear regression lines (blue) and confidence intervals (gray shading) plotted, showing associations between age (years) and adjusted rostro-caudal position measures (cm) (mean of left and right sides) for the maxillary cheek teeth in 67 horses. AdjMax06DiA, adjusted maxillary 06 disto-apical measurement; AdjMax06DiO, adjusted maxillary 06 disto-occlusal measurement; AdjMax11DiA, adjusted maxillary 11 disto-apical measurement; AdjMax11DiO, adjusted maxillary 11 disto-occlusal measurement. Negative measurements occur when the Triadan 11s are caudal to the orbit.

**Figure 7:**
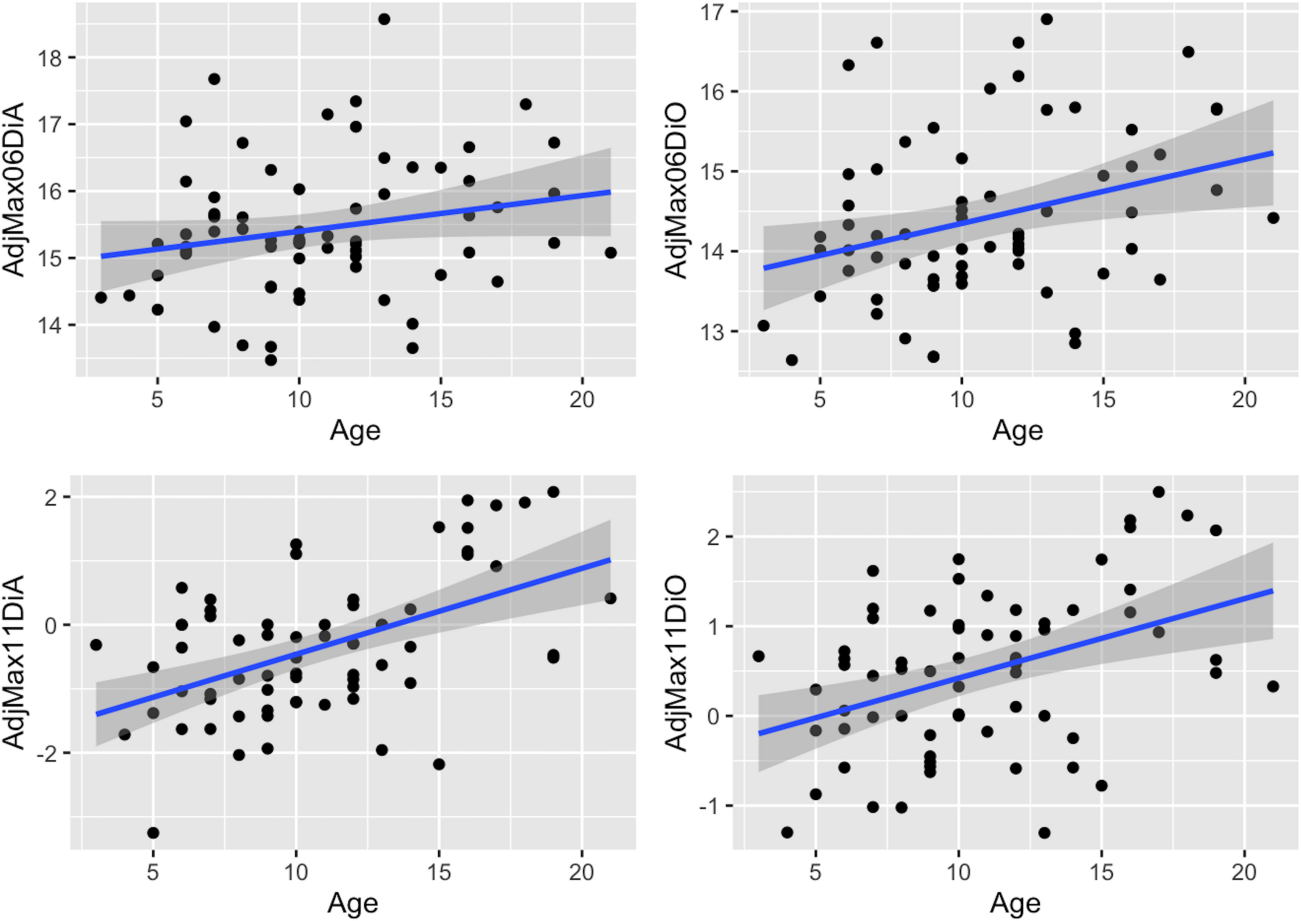
Scatterplots with linear regression lines (blue) and confidence intervals (dark gray shading) plotted, showing associations between age (years) and adjusted rostro-caudal position measures (mean of left and right) for the mandibular teeth in 67 horses. AdjMand06DiO, adjusted mandibular 06 disto-occlusal measurement; AdjMand06DiA, adjusted mandibular 06 disto-apical measurement; AdjMand11DiO, adjusted mandibular 11 disto-occlusal measurement; AdjMand11DiA, adjusted mandibular 11 disto-apical measurement.

**Figure 8:**
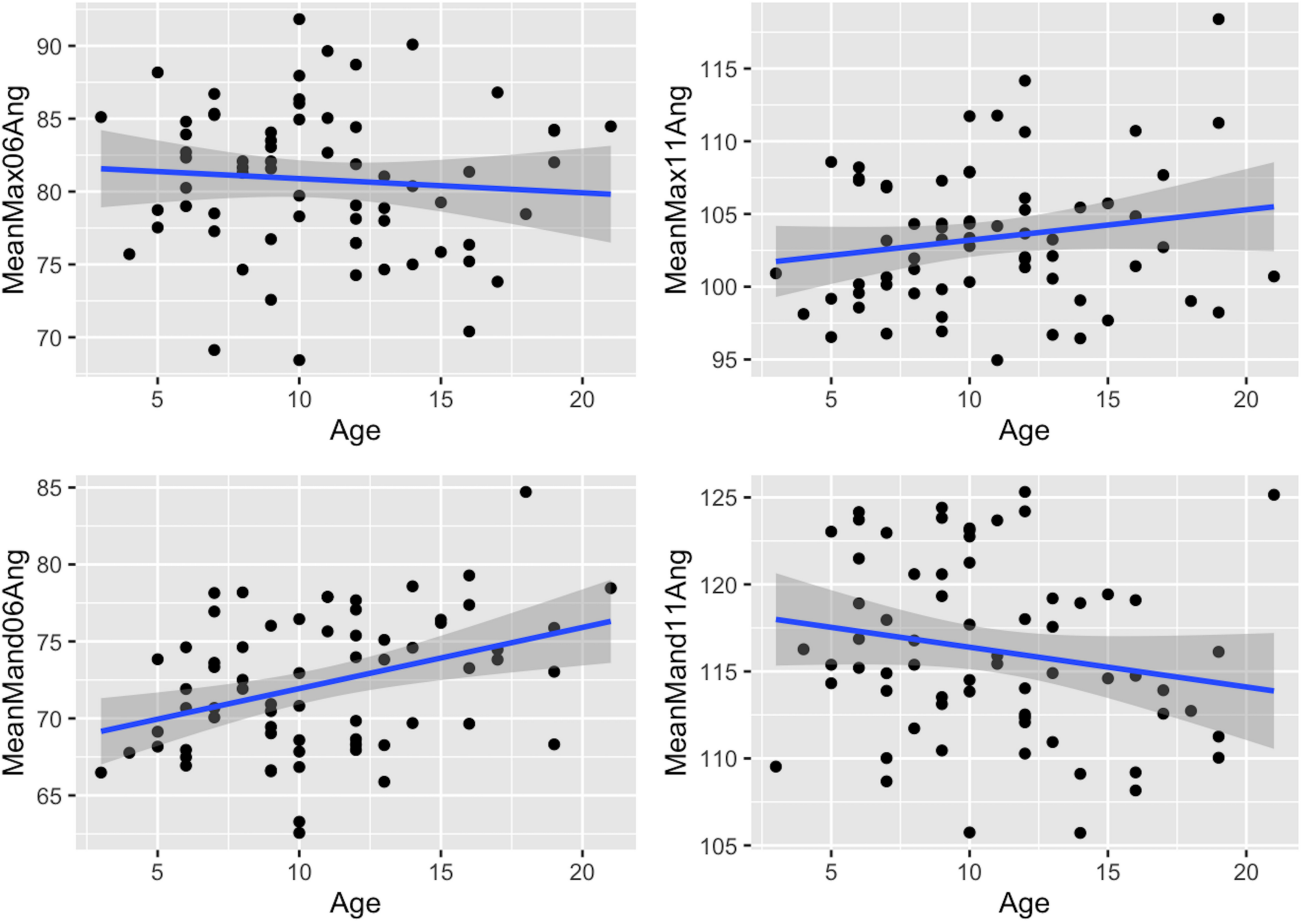
Scatterplots with linear regression lines (blue) and confidence intervals (dark gray shading) plotted, showing associations between age (years) and crown angles (mean of left and right) of the Triadan 06 and 11 teeth in 67 horses. MeanMax06Ang, maxillary 06 angle; MeanMax11Ang, maxillary 11 angle; MeanMand06Ang, mandibular 06 angle; MeanMand11Ang, mandibular 11 angle.

**Figure 9:**
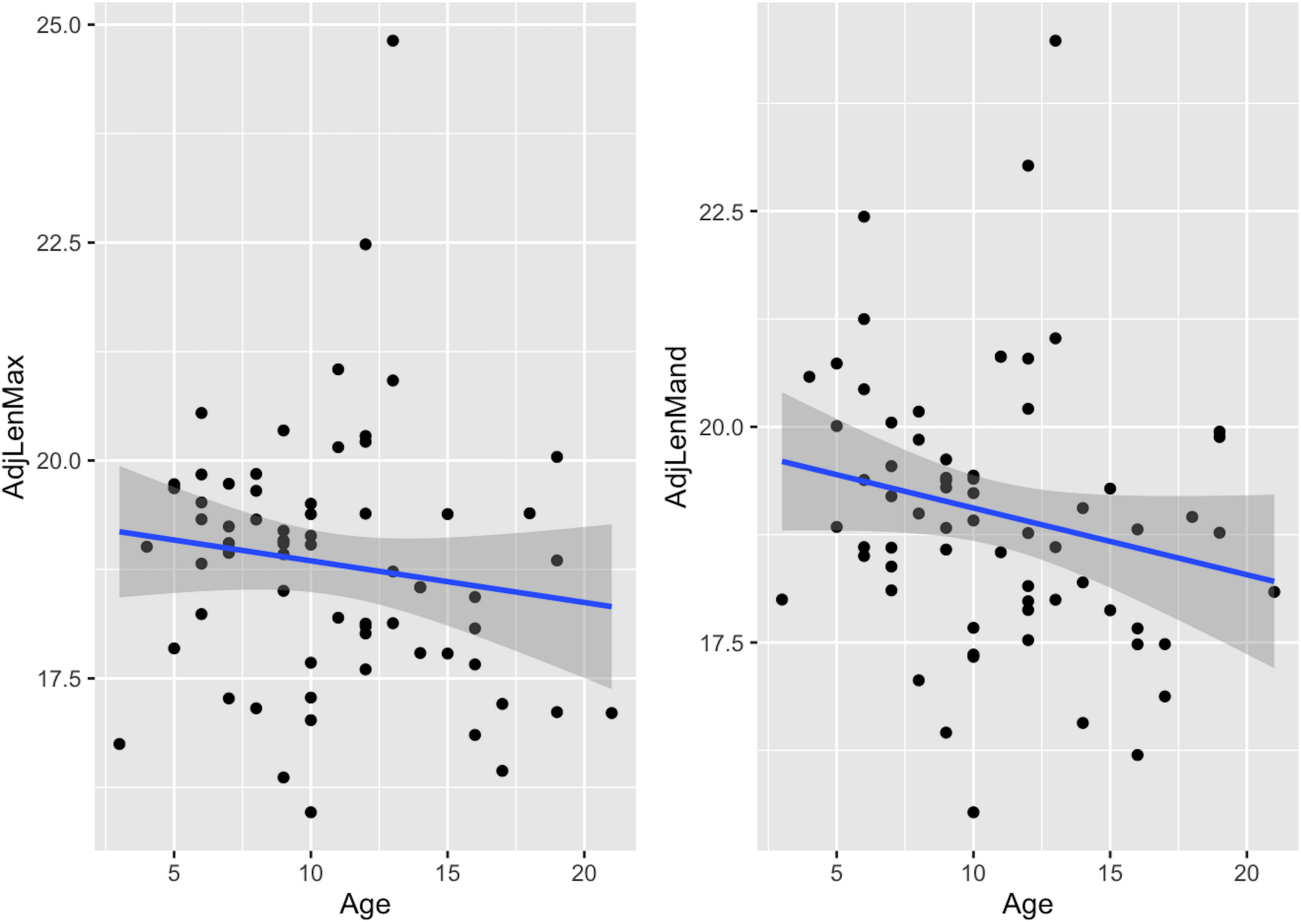
Scatterplots with linear regression lines (blue) and confidence intervals (dark gray shading) plotted, showing associations between age (years) and adjusted cheek teeth arcade lengths (cm) in 67 horses. AdjLenMax, adjusted mean maxillary cheek teeth arcade length; AdjLenMand, adjusted mean mandibular cheek teeth arcade length.

Associations between age and adjusted measures as well as dental angles are shown in Table 2 and Figures 6–**9**. These associations were not simply linear with much variation across the population, reflected in the correlation coefficients, which range from low to moderate correlation.

### Dental Drift

Measures of the apical and occlusal aspects of maxillary and mandibular 06 and 11s (Table 2 and Figures 6, 7) show the presence of age-related changes, including a moderate correlation in maxillary 06 disto-occlusal measure (0.32), maxillary 11 disto-apical and disto-occlusal measures (0.5 and 0.41, respectively), mandibular 11 disto-occlusal and disto-apical measures 0.37 and 0.46, respectively), mandibular 06 angle (0.37) and low correlation in maxillary 06 disto-apical and mandibular 06 disto-occlusal measures (0.22 and 0.26, respectively), mandibular 06 disto-apical measure (0.14), the maxillary and mandibular 11 angles (0.18 and −0.18, respectively), and the maxillary 06 angle (−0.08) and the maxillary and mandibular cheek teeth arcade (row) lengths (−0.14 and −0.21, respectively).

There was significant positive association between age and the adjusted disto-apical and disto-occlusal measures for the maxillary 11 and mandibular 11 teeth as well as with the adjusted disto-occlusal measure for the maxillary and mandibular 06 teeth and the mandibular 06 angles, indicating that both the 06 and 11 cheek teeth move mesially with age.

### Dental Angulation

The mandibular Triadan 06s (mean 17.8° off vertical) and 11s (mean 26.2°) had almost double the angulation of their maxillary 06 (mean 9.2°) and 11 (mean 13.3°) counterparts (Table 2 and Figure 8). Additionally, in both maxillary and mandibular teeth, the 06 angulation was lower than the Triadan 11 angulation (69 and 68%, respectively, for maxillary 11s and mandibular 11s). Age-related decreases in angulation were found in mandibular and maxillary 06 and 11s but were significant only in the mandibular 06 teeth.

### Arcade Lengths

Although both the maxillary and mandibular cheek teeth arcade lengths decreased with age (Table 2 and Figure 9), this decrease was not statistically significant in either cheek teeth row.

## Discussion

This study confirms the presence of age-related mesial drift of the caudal (Triadan 11s) equine maxillary cheek teeth previously quantified by Liuti et al. (3) with a mesial drift of 9 mm found between the <6 y.o. group and the 6–15 y.o. group and of 15 mm between the former and the >16 y.o. group in this study. Unexpectedly, the apical aspects of these teeth drifted slightly less (12 mm between the <6 and >16 y.o groups) as it was expected that their caudally facing apices would shorten as well as drift and, thus, overall have a higher measure of mesial drift than the occlusal aspects as was found by Liuti et al., who recorded drift of 24.8 mm in the occlusal surface and 28.3 mm in the maxillary Triadan 11s of similar age groups of horses.

Age-related mesial drift has not been reported in maxillary Triadan 06s. This study shows a mean mesial drift of 15 and 12 mm, respectively, in the occlusal and apical aspects of maxillary 06s between the <6 and >16 y.o groups. The apical aspects of these mesially facing apices drifted slightly less as expected as they became short as well as drifting mesially.

Equine mandibular cheek teeth dental drift has also not previously been documented. Similarly to the maxillary cheek teeth, there was an increased level of mesial drift of the distally facing apices (26 mm) as compared with their occlusal aspects (18 mm) of the mandibular Triadan 11 teeth (Table 3) because of a mesially directed eruption of these apices as well as mesial dental drift. For similar reasons, there was *less* drift of the apices of the mandibular 06s (4 mm) compared with their occlusal aspects (13 mm) between the youngest and oldest age groups.

The age-related movement of the Triadan 06s is more complex to understand because of the competing forces of mesial drift induced by the angulation of the Triadan 11 (and possibly by the variably angulated Triadan 10 teeth) and the distally directed forces resulting from the (angulated) Triadan 06 teeth themselves.

Following extraction of teeth within the equine cheek teeth row, the anterior teeth drift distally and also incline into the extraction site (9–11). However, the net result of normal age-related, posteruption movements found in this study was a mesial drift of the Triadan 06s (maxillary 15 mm, mandibular 13 mm) of similar magnitude to the Triadan 11s (maxillary 18 mm, mandibular 15 mm) (Table 3).

These finding indicate that the mesially directed forces are greater than the distally directed forces. This may be explained by the much greater angulation of both the mandibular and maxillary 11s (mean of all of horses of 26.2° and 13.3°, respectively) compared with the lower and upper 06s (17.8° and 9.2°, respectively) and also because the occlusal pressures are highest more caudally in the oral cavity.

It was hypothesized that there would be an age-related decrease in cheek teeth angulation with age, but there was significant age-related change in angulation only in the mandibular 06 teeth (decreased from 20.9° in the youngest to 14.7° in the oldest group) and a non-significant decrease in the mandibular 11s (from 25.7° to 23.9°). However, in the maxillary cheek teeth, the angles actually increased (from 8.9° to 11.2° in the 06s and from 10.7° to 15.4° in the 11s) between the youngest and oldest groups. The maintenance of adequate cheek teeth angulation appears to be pivotal in maintaining tight interdental contact in teeth, and so it should not be surprising that adequate angulation was maintained.

Limitations in these cheek teeth angulation measurements include that these angles were taken as the line of best fit of the clinical and more occlusal aspect of the reserve crown rather than from the more angulated apical aspects of these teeth with curved roots, a feature of older mandibular cheek teeth. With more advanced age-related wear, some 06 and 11 angulations are fully lost when the reserve crowns become very short with wear.

Examination of the association between the cheek teeth row lengths and horse age showed a non-significant trend toward reduction with age from a mean of 18.6 mm long on the youngest to 17.9 mm in the oldest group in the maxillary cheek teeth and from 19.6 to 18.2 mm long in the mandibular arcade. Examination of the clinical crowns of equine cheek teeth shows the obvious presence of interdental wear, including the complete loss of peripheral cementum at these sites. This interproximal wear in conjunction with the tapering crowns of equine teeth results in shorter cheek teeth rows in older horses. If larger numbers of horses were examined, these measurements may have become statistically significant. Limitations on these measurements include the variable curvature of the equine cheek teeth occlusal surface, including, more caudally, the curve of Spee.

Another limitation is the small number of horses included in this study. Despite having collated 358 studies of horses that underwent standing head CT, 291 were removed due to motion artifact during image acquisition, missing or supernumerary teeth, cheek teeth diastemata, displaced or fractured cheek teeth, or craniofacial abnormalities (e.g., brachygnathism or prognathism). It was considered important to use these strict exclusion criteria to remove the potential impact of these factors on the measurements.

The development of mobile platforms that allow CT imaging in standing sedated horses is a major improvement with reduced costs and morbidity/mortality risks when compared with general anesthesia as previously noted (16, 17). However, motion artifact is commonly seen during image acquisition, and it is considered the main cause of poor image quality (18).

From a clinical perspective, this study shows that, in general, horses maintain the occlusal angulations of their cheek teeth well into their adulthood. This angulation appears to be the prime factor in keeping the cheek teeth occlusal surfaces in close contact and preventing diastemata from developing. Overall, the mesial drift caused by the caudal cheek teeth, especially the 11s, is stronger than the distal drift induced by the 06s, resulting in an overall mesial drift while keeping all occlusal surfaces together. For these angulated teeth to be effective in causing both mesial and distal dental drift, adequate occlusal forces must be applied to them, and thus, relieving dental pain by treating periodontal disease would appear useful. Likewise, all teeth should be free to drift, and thus, removal of any occlusal overgrowths that may restrict such drift is also essential.

## Conclusions

In the population of horses used for this study, age-related mesial drift occurred in both Triadan 06 and 11s, and the angulation of these teeth also decreased with age. A study with a larger number of cases with more age variability and/or following horses over their lives would be useful to better understand the development of certain conditions such as diastemata.

## Data Availability Statement

The original contributions presented in the study are included in the article/supplementary material, further inquiries can be directed to the corresponding author.

## Ethics Statement

This study was approved by the Ethical Committee of The University of Edinburgh Veterinary School (52.21). Written informed consent was obtained from the owners for the participation of their animals in this study.

## Author Contributions

TL, PD, and RR contributed to conception, design of the study, and wrote sections of the manuscript. TL and CD organized the database. RR performed the statistical analysis. PD wrote the first draft of the manuscript. All authors contributed to manuscript revision, read, and approved the submitted version.

## Conflict of Interest

The authors declare that the research was conducted in the absence of any commercial or financial relationships that could be construed as a potential conflict of interest. The handling editor declared a past co-authorship with one of the authors PD.

## Publisher's Note

All claims expressed in this article are solely those of the authors and do not necessarily represent those of their affiliated organizations, or those of the publisher, the editors and the reviewers. Any product that may be evaluated in this article, or claim that may be made by its manufacturer, is not guaranteed or endorsed by the publisher.
